# A Medical Causerie

**Published:** 1916-04-22

**Authors:** 


### A MEDICAL CAUSERIE.

The attacks on the Central Medical War Committee made an energetic showing for a time, but the logic of facts has been too much for them. The alternative scheme which was to be produced, and for which practitioners were invited to wait by a letter in the *Times*, was in a second letter withdrawn. Again, the contention that the Army already had doctors and to spare has been strangled by the direct contradiction of the Director-General. In the meantime the Committee has gone on with its work, and it may be hoped that ere long we shall have some definite announcement of the degree of its success. From the Scottish Committee there is an intimation that Scotland may certainly be relied on to supply the number of medical officers which fall to its share. An aspect of the subject which has not received much attention is the possibility that more surgeons may be required by the Navy. For the time being it is understood that the senior Service has a full equipment, but obviously circumstances may arise in which more help will be needed, and such a claim would clearly take precedence of all others.

---

A proposal which was mooted some months ago has again come to the front—namely, the institution of a professional fund having some relation to the position of civil practitioners who have given up their practices under the stress of the national emergency to take service in the R.A.M.C. The Central Committee has now asked the Council of the British Medical Association to consider the advisability of establishing such a fund. In the Press announcements which have appeared on the subject the proposed fund is spoken of as a "compassionate" one. But it is to be hoped that this will not be the official title. Such a term would certainly not appeal to applicants, nor would it be likely either to invite subscribers. What is wanted is some tangible evidence from the profession of the sympathy with which they regard the action of members who have accepted the risk of personal and professional loss for the sake of the nation's interest. In some cases this might certainly take a financial shape, and in all it might appear in the form of a record which would be a legitimate source of pride to the owner. The idea is to witness to the officers themselves and to the public that the whole profession stands together in this matter. Presumably there will be in time an official war medal which everyone who has been on active service will be entitled to receive. But a definite professional recognition would, of course, have peculiar value, and it is to be hoped that one of suitable character will be arranged. The question of money compensation will be one of great difficulty, and, in many instances at least, can hardly be attempted. But a professional fund, if it can be established, would have much sentimental value, and in individual instances might help to meet a position which has a severe practical aspect.

Some apprehension has been expressed in medical circles lest the new College of Nursing may tend to introduce differences where union is essential, and an official statement on the point from the organisers of the College might be helpful. No one wants to hinder any measure which will promote efficiency in nursing and will further the interests of individual nurses. But whatever height these may attain, it must always remain true that nursing activities must be conducted in subordination to medical directions, and this in the interests not of the doctors, but of the patients. The responsibility and the knowledge in each individual case are with the practitioner, and with him, therefore, necessarily must rest full authority. Any increase of skill and effectiveness and intelligence on the part of those who carry out medical orders, whether nurses or others, is, of course, to be welcomed, but there can be no division of authority. Doubtless those concerned with the establishment of the College fully realise this necessity, but in some quarters a different tone has been heard. A clear and authoritative statement on the point would help to allay apprehensions, and would secure support which may otherwise be withheld. The hearty co-operation of the medical profession would be an asset of great value, and it can probably be secured if the point here indicated receives due attention and emphasis.

---

The fashion of proclaiming how much more excellent are German methods than those practised in our own country has lost something of its vogue of recent months, so far at least as national activities in peace are concerned. A somewhat belated attempt to revive it has recently been made by the medical correspondent of the *Times*, who, writing on the increase of population in Germany, announced to his readers that the growing total was due not merely to the number of births, but also to the "prevention of infant death, which is one of the greatest works standing to the credit of a scientific people." Such a statement must have excited something more than surprise in the minds of those who know anything about the subject, and it was promptly challenged by Dr. Brend, who shows that the infant mortality rate in Germany is among the worst in Europe. The figures from 1901 to 1912 announce an average infant mortality of 184 per 1,000 births in Germany, as compared with 121 in the United Kingdom. Our tables are bad enough in all conscience, but it is clear we are not as black as we are painted—by our friends. Resolute efforts are being made to reduce a loss which the public cannot fail to see is a source of national weakness, and even of danger. The magnitude of the evil is pretty clearly comprehended now by many thoughtful persons outside the ranks of the medical profession; and if only the question escapes the taint of party politics there is a prospect of real progress in the near future.

